# Effects of transfusion load and suction pressure on renal function in intraoperative salvage autotransfusion

**DOI:** 10.1590/1414-431X202010292

**Published:** 2021-01-15

**Authors:** Jingyang Zeng, Sijie Zhang, Qilin Wu, Shunyuan Li, Yingle Chen, Biyu Wu

**Affiliations:** 1 Quanzhou First Hospital Affiliated to Fujian Medical University, Department of Anesthesiology, QuanzhouFujian China Department of Anesthesiology, Quanzhou First Hospital Affiliated to Fujian Medical University, Quanzhou, Fujian, China; 2 Graduate School of Fujian Medical University, FuzhouFujian China Graduate School of Fujian Medical University, Fuzhou, Fujian, China; 3 Quanzhou First Hospital Affiliated to Fujian Medical University, Department of Nursing, QuanzhouFujian China Department of Nursing, Quanzhou First Hospital Affiliated to Fujian Medical University, Quanzhou, Fujian, China

**Keywords:** Intraoperative salvage autotransfusion, Plasma-free hemoglobin, Creatinine clearance

## Abstract

Although some investigations have been performed to determine the effects of transfusion load and suction pressure on renal function during intraoperative salvage autotransfusion, the precise threshold is still undetermined. A total of 625 patients undergoing surgery with the Continuous AutoTransfusion System (CATS^plus^) were enrolled and divided into groups according to the utilized suction pressure and transfusion volume. Plasma free hemoglobin (FHB) and creatinine clearance (CCr) were assayed to indicate the renal function. Both 0.03 MPa suction (≥4-unit load) and >5 units transfusion changed the levels of FHB and CCr significantly when measured 24 h post-operation compared to pre-operation. Under 0.02 MPa suction (≥4-unit load), the alteration of FHB and CCr returned to normal after 24 h. Under 3 units transfusion, the levels of FHB and CCr at 6 and 12 h post-operation changed significantly compared to pre-operation (P<0.05 or P<0.01, respectively), and this alteration could be restored to normal at 72 h post-operation. After an exhaustive investigation, less than 4 units transfusion and less than 0.03 MPa suction pressure are recommended for intraoperative salvage autotransfusion.

## Introduction

Besides financial burden, allogenic transfusion remains a challenge considering the risk of developing transfusion-transmitted virus and bacterial infections, acute lung injury and circulatory overload, hemolytic and allergic reactions, variant Creutzfeldt-Jakob disease, and even death (1–4). Thus, intraoperative blood salvage should be considered. With carefully selected surgical patients, intraoperative salvage autotransfusion can provide erythrocytes of a higher quality with less blood storage and immunological challenge (5,6). Considering the risks, intraoperative salvage autotransfusion may be more cost-effective than allogeneic transfusion. The global medical community has increasingly moved from allogeneic towards autologous blood transfusions.

Intraoperative salvage autotransfusion is reported to affect post-operative renal function (7,8). In the process of retrieving and transfusion of hematochezia, erythrocytes are sucked, filtered, and separated by vacuum aspiration, which will cause some degree of hemolysis-related deterioration of renal function. In order to achieve maximum clinical benefit and minimum adverse effect, the negative suction pressure, the size of the suction tip, and the air contact during suction were investigated in a previous report, and it is estimated that air contact is a major concern (9). The Fresenius Kabi Continuous AutoTransfusion System is standardly utilized to aspirate in the middle of the pool of blood to minimize air contact (10), and the balance between the volume of transfusion and vacuum suction pressure was assessed in that investigation. In order to minimize the mechanical breakage of recycled erythrocytes, the vacuum suction pressure is recommended to not exceed 0.02 MPa (150 mmHg) by previous research (11), while others found that erythrocytes are not affected even at 0.04 Mpa (12). It is believed that the concentration of free hemoglobin (FHB) in the recovered blood is positively correlated with the occurrence of hepato-renal function insufficiency, and up to 1 g/L FHB can cause renal function damage. More than five units of autologous blood transfusion could increase the incidence of renal failure after surgery.

This investigation was designed to comprehensively evaluate the effect of transfusion volume and suction pressure on renal function, which will help to set up a unified standard of intraoperative salvage autotransfusion.

## Material and Methods

### Study subjects

This investigation was performed in Quanzhou First Hospital of Fujian Province, and the protocols were approved by the Ethical Committee of Quanzhou First Hospital. Study design and procedures are shown in Figure S1. A total of 936 typical cases that underwent surgery using the Continuous AutoTransfusion System (CATS^®plus^, Fresenius Kabi, Germany) were screened from January 2016 to December 2018. Inclusion criteria were: over 18 years of age; willing to participate and sign an informed consent; and underwent orthopedic surgery, cardiovascular surgery, organ transplant surgery, brain surgery, emergency surgery, and other surgeries. Exclusion criteria were: surgical patients with comorbidities such as hypertension, diabetes mellitus, chronic obstructive pulmonary disease, and chronic renal disease; surgical field blood contaminated with digestive, bile, urine, and amniotic fluid; surgical field blood at risk of contamination in open trauma; blood conservation beyond 6 h; patients with infectious diseases; patients with malignant tumors and those who died during or within 72 h after surgery; patients with kidney injury before operation. After evaluation, a total of 625 eligible patients were enrolled in the final investigation.

### Continuous autotransfusion procedure

The CATS^plus^ circuit was primed and purged with 3000 mL saline (0.9%) mixed with 30,000 IU of sodium heparin. A double-lumen suction catheter specific for blood collection was utilized to salvage the bloodshed from the surgical field. Salvaged blood was suctioned with different vacuum pressures according to the study design (0.01, 0.015, 0.02, and 0.03 MPa), and further collected into the reservoir of the CATS^plus^. High-quality wash performed with the CATS^plus^ was processed to minimize the residual FHB (<100 mg/dL), and the separated erythrocytes were further collected and transfused into the patients. Residual heparin levels were guaranteed to be less than or equal to 0.1 IU/mL in all post-treatments to minimize the clinical risk for intra- or post-operative hemorrhage.

### Demographical and hematological variables

Age, gender, body mass index (BMI), and smoking habit were obtained and recorded. Hematological variables, such as blood urea nitrogen (BUN), blood creatinine, aspartate aminotransferase (AST), alanine aminotransferase (ALT), plasma FHB, and creatinine clearance (CCr), were measured.

### Statistical analysis

GraphPad Prism v7.0 software (GraphPad Software, Inc., USA) was used to analyze the data. Numerical variables are reported as means±SD. Categorical data are reported as percentage (%) and were compared between groups by Pearson chi-squared and Fisher's Exact tests. Because the data of FHB and CCr were not in a normal distribution (Kolmogorov Smirnov, P<0.05), the Mann Whitney test was used to test the statistical significance of FHB and CCr. A significance level of P<0.05 was used in all tests.

## Results

### Clinical characteristics

Preoperative data (BMI, serum BUN, blood creatinine, AST, and ALT) of participants involved in this investigation are reported in Table 1. Age, gender, smoking habit, as well as types of surgery are also indicated in Table 1. The participants were further randomly assigned to different groups according to the volume of blood transfusion (2, 3, 4, 5, and >5 units) and suction pressure (0.01, 0.015, 0.02, and 0.03 MPa) (Table 2).

**Table 1:** Characteristics of study subjects.

Variables	Mean±SD or number (%)
Gender	
Male	349 (55.8%)
Female	276 (44.2%)
Age	
<30 years	34 (5.4%)
30-45 years	137 (21.9%)
46-60 years	285 (45.6%)
>60 years	169 (27.1%)
BMI	
<18.5	16 (2.6%)
18.5-24.9	267 (42.7%)
25-30	321 (51.3%)
>30	21 (3.4%)
Smoking habit	127 (20.3%)
BUN (mg/dL)	16.4±6.3
Blood creatinine (mg/dL)	0.87±0.39
AST (IU/L)	37±15
ALT (IU/L)	54±36
Operation type	
Orthopedic surgery	105 (16.8%)
Cardiovascular surgery	123 (19.7%)
Organ transplant surgery	86 (13.8%)
Brain surgery	94 (15.0%)
Emergency surgery	101 (16.2%)
Others	116 (18.5%)

BMI: body mass index [<18.5 (low weight); 18.5-24.9 (normal); 25-30 (overweight), and >30 (obese)]; BUN: blood urea nitrogen; AST: aspartate aminotransferase, ALT: alanine aminotransferase.

**Table 2:** Subjects were assigned to different number of transfusion units and pressures of intraoperative salvage autotransfusion.

Suction pressure	2 units	3 units	4 units	5 units	>5 units
0.01 MPa	26 (4.2%)	29 (4.6%)	34 (5.4%)	19 (3.0%)	19 (3.0%)
0.015 MPa	37 (5.9%)	47 (7.5%)	38 (6.1%)	27 (4.3%)	18 (2.9%)
0.02 MPa	39 (6.2%)	53 (8.5%)	43 (6.9%)	36 (5.8%)	21 (3.4%)
0.03 MPa	42 (6.7%)	33 (5.3%)	24 (3.8%)	23 (3.7%)	17 (2.7%)

Data are reported as number (%).

### Transfusion load and suction pressure related to risk of renal function damage

Postoperative renal function change was indicated by the alteration of plasma FHB and CCr. A significant difference in FHB and CCr 24 h post-operation compared with pre-operation in the case of 0.03 MPa suction and ≥4 units transfusion and 0.02 MPa and >5 units transfusion (P<0.05 or P<0.01) (Table 3 and Table 4) was found, which indicated that both >5 units transfusion and 0.03 MPa suction plus ≥4 units transfusion would increase the risk of renal function damage.

**Table 3:** Plasma free hemoglobin (mg/dL) content in pre- and post-operative patients at 24 h.

Suction pressure	2 units	3 units	4 units	5 units	>5 units
0.01 MPa					
Pre-operation	7.73±5.21	8.91±6.31	6.32±4.98	9.21±6.12	7.12±5.61
Post-operation	15.16±7.23	21.47±7.93	26.05±10.42	31.11±14.65	45.65±12.34*
0.015 MPa					
Pre-operation	6.87±5.03	8.32±5.82	7.92±4.82	7.54±5.55	8.11±6.04
Post-operation	13.89±7.14	25.23±9.27	28.38±12.16	29.75±13.28	53.05±14.18*
0.02 MPa					
Pre-operation	9.14±6.31	8.27±5.72	7.31±5.19	7.79±6.05	7.38±5.23
Post-operation	16.27±7.12	28.12±13.11	32.65±15.21	48.92±13.27*	72.05±18.24*
0.03 MPa					
Pre-operation	6.94±4.90	7.67±5.81	8.81±5.94	9.03±5.92	7.49±4.94
Post-operation	30.73±11.79	42.87±13.29*	62.15±18.57*	87.34±25.12*	121.28±33.73**

Data are reported as means±SD. *P<0.05, **P<0.01 compared to pre-operation at the same condition during the intraoperative salvage autotransfusion (paired *t*-test).

**Table 4:** Creatinine clearance (mL·min^-1^·(1.73 m^2^)^-1^) in pre-operative and 24 h post-operative patients.

Suction pressure	2 units	3 units	4 units	5 units	>5 units
0.01 MPa					
Pre-operation	92±7	89±8	90±7	93±10	88±9
Post-operation	84±9	83±10	80±10	76±10	45±12*
0.015 MPa					
Pre-operation	89±10	91±9	88±11	90±9	91±9
Post-operation	81±9	76±10	77±12	70±13	47±14*
0.02 MPa					
Pre-operation	92±10	86±11	89±10	94±13	91±11
Post-operation	83±12	74±13	71±12	63±9*	42±10*
0.03 MPa					
Pre-operation	89±13	90±9	88±7	91±12	89±9
Post-operation	72±10	69±12	60±11*	51±10*	35±9**

Data are reported as means±SD. *P<0.05, **P<0.01 compared to pre-operation at the same condition during intraoperative salvage autotransfusion (paired *t*-test).

### Less than 4 units of transfusion volume caused no harm to renal function

To decipher the relative long-term effect of transfusion volume and suction pressure on the risk of developing renal function damage, a time-course detection and analysis were performed. Under the situation of 0.02 MPa suction (>5 units transfusion), the levels of FHB and CCr altered significantly compared with the pre-operation condition at 6, 12, 24, and 72 h, as expected (P<0.05 or P<0.01, Table 5). Three units transfusion caused no change in FHB and CCr, while the change of FHB and CCr returned to normal after 24 h post-operation with 4 units transfusion, and all of these indicated that a transfusion volume less than four units should be safe.

**Table 5:** Plasma free hemoglobin (FHB) and creatinine clearance (CCr) in pre-operative and 6, 12, 24, and 72 h post-operative patients under 0.02 Mpa pressure.

	Preoperative	6 h	12 h	24 h	72 h
FHB, mg/dL					
2 units	9.14±6.31	32.36±16.27	24.47±7.62	16.27±7.12	10.34±6.51
3 units	8.27±5.72	36.91±10.85	30.61±10.79	28.12±13.11	20.16±9.42
4 units	7.31±5.19	43.68±12.37*	34.95±11.64	32.65±15.21	22.39±9.94
5 units	7.79±6.05	113.61±20.33**	75.77±17.72*	48.92±13.27*	31.75±14.12
>5 units	7.38±5.23	135.42±26.87**	106.45±19.85**	72.05±18.24*	53.39±16.11*
CCr, mL·min^-1^·(1.73 m^2^)^-1^					
2 units	92±10	75±13	78±9	83±12	90±14
3 units	86±11	71±11	72±11	74±13	88±9
4 units	89±10	63±10*	65±9*	71±12	83±10
5 units	94±13	39±10*	46±12*	63±9*	67±10*
>5 units	91±11	36±12*	39±9*	42±10*	53±11*

Data are reported as means±SD. *P<0.05, **P<0.01 compared to pre-operation at the same condition during intraoperative salvage autotransfusion (paired *t*-test).

### Suction pressure less than 0.03 MPa caused no harm to renal function

The effect of suction pressure on renal function was further investigated under the 3 units transfusion. The changes of FHB and CCr at 6 and 12 h post-operation was significant compared with pre-operation (P<0.05 or P<0.01), while such changes were restored to normal at 72 h post-operation at 0.03 MPa suction (Table 6). Only at the 6-h time-point was a FHB change observed at 0.02 MPa suction, which was restored at 12 h post-operation. All of these indicated that the recommended pressure for vacuum suction should be less than 0.03 MPa.

**Table 6:** Plasma free hemoglobin (FHB) and creatinine clearance (CCr) in pre-operative and 6, 12, 24, and 72 h post-operative patients under 3 transfusion units.

Suction pressure	Preoperative	6 h	12 h	24 h	72 h
FHB, mg/dL					
0.01 MPa	8.91±6.31	31.65±14.48	24.36±12.29	21.47±7.93	13.52±9.11
0.015 MPa	8.32±5.82	35.14±13.95	28.24±14.17	25.23±9.27	16.86±8.42
0.02 MPa	8.27±5.72	64.97±15.26*	34.75±16.63	28.12±13.11	21.72±9.78
0.03 MPa	7.67±5.81	98.86±18.56**	68.25±14.47*	42.87±13.29*	35.25±14.17
CCr, mL·min^-1^·(1.73 m^2^)^-1^					
0.01 MPa	89±8	73±13	79±14	83±10	83±15
0.015 MPa	91±9	70±12	73±11	76±10	85±9
0.02 MPa	86±11	68±15	75±16	74±13	81±13
0.03 MPa	90±9	46±14*	52±12*	69±12	73±13

Data are reported as means±SD. *P<0.05, **P<0.01 compared to pre-operation at the same condition during intraoperative salvage autotransfusion (paired *t*-test).

## Discussion

In this investigation, both FHB and CCr were utilized to indicate renal function. Intraoperative salvage hemolysis can release FHB, which can precipitate in the renal tubules with resultant tubular dysfunction (13–15). In intraoperative salvage autotransfusion, the washing process can remove FHB before re-infusion, and less than 100 mg/dL FHB is recommended to minimize the effect on renal function. Such concentration of FHB is well below the standard of banked blood, which will show no deleterious effects on renal function after transfusion. CCr is a parameter that can be utilized to assess the excretory function of the kidneys (16–18), and renal dysfunction can be defined as a decrease of CCr (<60 mL/min) (19).

Intraoperative salvage autotransfusion performed by the CATS^plus^ system has some advantages, such as higher oxygen-carrying capacity and lower volume requirement for transfusion. The efficiency of erythrocyte collection during cell salvage can be attributed to suction pressure. Previous investigations suggest that minimal suction pressures and a suction tip with a relatively large diameter should be adopted to aspirate blood from a surgical field to minimize blood-air interfaces and maximize the efficiency of erythrocyte recovery (11,20). The fast-moving foams produced by air aspiration during blood suction will burst in the negative pressure environment and generate a mechanical shear force on the erythrocyte (9,21,22). In this investigation, we found that a suction pressure of less than 0.03 MPa should be used for orthopedic surgery, cardiovascular surgery, organ transplant surgery, brain surgery, and emergency surgery, which can help guide the clinical application of intraoperative salvage autotransfusion. Minimum initial blood volume is required by the CATS^plus^ system to process; in this research, less than four units transfusion was determined for minimal renal function damage (23,24).

The introduction and development of minimally invasive surgery will limit the utilization of intraoperative salvage autotransfusion due less blood loss and transfusion requirements (25–27). Considering cost-to-benefit ratios, such as lower infection rates and shorter hospital stays, intraoperative salvage autotransfusion can still benefit several patients. In addition, allogeneic transfusion, but not autologous transfusion, could shift an immune deviation towards a T helper 2-type response. Such a shift is associated with a gene transcription profile characteristic of immunosuppression, which may suppress natural killer cells and cytotoxic T-cells, and activate regulatory T cells to down-regulate cellular immunity (25,28,29). Although the long-term immune shifting effect still needs further detailed analysis, intraoperative salvage autotransfusion might be a safe option in the immune context. In addition to renal function, the influence of intraoperative salvage autotransfusion on proinflammatory cytokines, platelet activation, coagulation, fibrinolysis, and hemolysis factors should also be considered in clinical practice, as reported (10,30,31), which will be analyzed in a future clinical study. All of these indicate that in addition to the more detailed evaluation of renal function by glomerular filtration rate and blood urea nitrogen, immune function change induced by intraoperative salvage autotransfusion should also be studied.

It must be mentioned that prolonged large-volume autotransfusion may cause coagulopathy due to the dilution of clotting factors with transfusions. Thus, together with appropriate transfusion load and suction pressure, regular coagulation function or close patient monitoring is required (32,33). The long-term effect of the tested transfusion load and suction pressure on renal function was not investigated in this study, and such limitation should also be considered.

Our investigation has both scientific and practical implications for the utilization of intraoperative salvage autotransfusion, which will help clinicians prescribe the appropriate amount of autotransfusion volume and suction pressure.

## Conclusion

Although a more precise design and multi-center investigation are needed, this study showed that less than four units transfusion and less than 0.03 MPa suction pressure may be recommended for intraoperative salvage autotransfusion.

## Supplementary Material

Click here to view [pdf].
